# Phytoplankton abundance and structural parameters of the critically endangered protected area Vaya Lake (Bulgaria)

**DOI:** 10.1080/13102818.2014.947718

**Published:** 2014-10-31

**Authors:** Ralits Dimitrova, Elena Nenova, Blagoy Uzunov, Maria Shishiniova, Maya Stoyneva

**Affiliations:** ^a^ Department of Botany, Faculty of Biology, Sofia University “St. Kliment Ohridski”, Sofia, Bulgaria; ^b^ Department of Zoology and Anthropology, Faculty of Biology, Sofia University “St. Kliment Ohridski”, 1164Sofia, Bulgaria

**Keywords:** numbers, biomass, carbon content, diversity, evenness, dominance, cyanoprokaryotes

## Abstract

Vaya (Ramsar site, protected area and Natura 2000 site) is the biggest natural lake in Bulgaria and the shallowest Black Sea coastal lake, which during the last decades has undergone significant changes and was included as critically endangered in the Red List of Bulgarian Wetlands. Our studies were conducted during the summer and autumn months of three years – 2004–2006. The paper presents results on the phytoplankton abundance (numbers, biomass and carbon content) in combination with the indices of species diversity, evenness and dominance. Phytoplankton abundance was extremely high (average values of 1135 × 10^6^ cells/L for the quantity and of 46 mg/L for the biomass) and increased in the end of the studied period (years 2005–2006), when decrease of species diversity and increase of the dominance index values were detected. The carbon content of the phytoplankton was at an average value of 9.7 mg/L and also increased from 2004 to 2006. Cyanoprokaryota dominated in the formation of the total carbon content of the phytoplankton, in its numbers (88%–97.8%), and in the biomass (62%–87.9%). All data on phytoplankton abundance and structural parameters in Vaya confirm the hypertrophic status of the lake and reflect the general negative trend in its development.

## Introduction

Vaya ( = Burgas Lake) is the biggest natural lake in Bulgaria and the shallowest Black Sea coastal lake. Vaya is a part of the large wetland complex of several Burgas lakes (including Atanasovsko Lake and Mandra Dam), located on the ‘Via Pontica’ ornithological migratory route and is important for the conservation of rare and endangered species of national, European and global significance. The lake is a Ramsar site, a protected area in the Bulgarian legislation and the national ecological network Natura 2000. During the last decades the aquatic ecosystem of Vaya has undergone significant changes due to different anthropogenic factors. The main among them are the disturbance of the water balance and the introduction of biogenic elements in the lake, combined with decreased halinity, which have a negative effect on the hydrochemical composition of the water and its flora and fauna. Therefore it has been included as critically endangered in the Red List of Bulgarian Wetlands.[1,2]

Complex study of the lake hydrobiology, carried out in 2004–2006 [3] forerunned in October 2003 by hydrochemical studies,[4] include phytoplankton investigation, a part of which, concerning its taxonomic structure, is published elsewhere.[5] In the present study the quantitative characteristics of the Vaya phytoplankton (numbers, biomass and carbon content) and its key structural indices (of species diversity, evenness and dominance) in the changed conditions of the lake due to the anthropogenic impact, summarized in Michev and Stoyneva [2], are presented. A comparison of our results with those from other phytoplankton investigations [5–9] is made.

## Materials and methods

**Figure 1.**
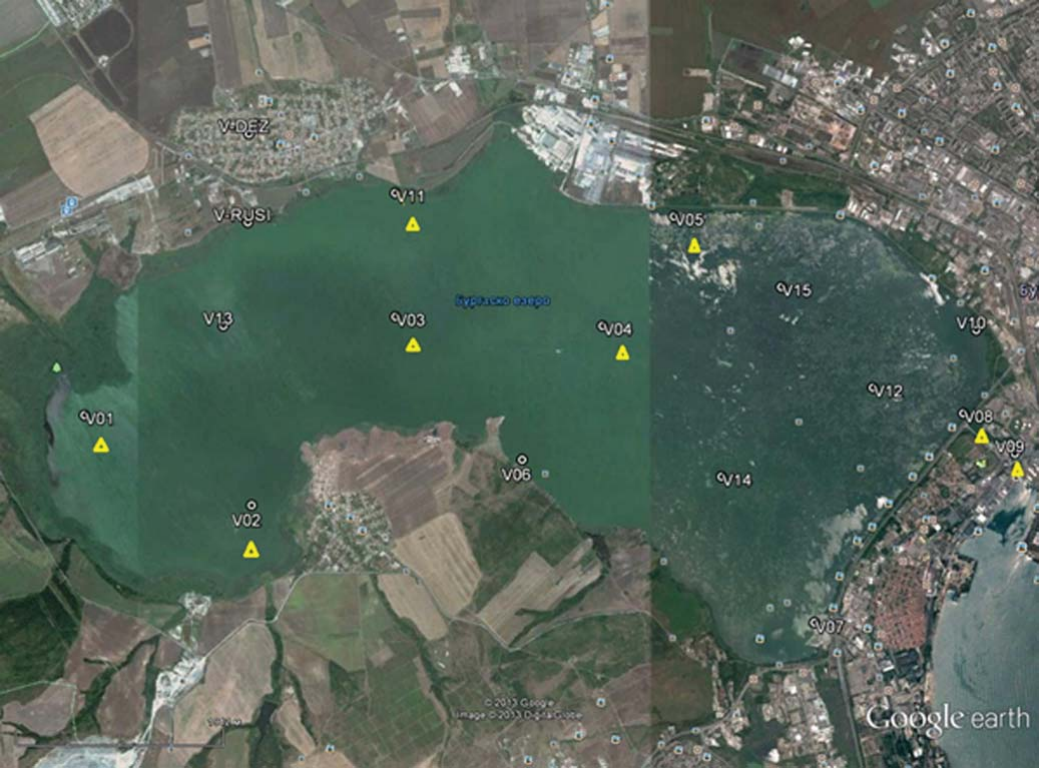
Map of Vaya Lake with sampling points.

The study with the focus on the phytoplankton was conducted during the summer and autumn months of three consecutive years – 2004, 2005 and 2006, as a part of a more complete hydrobiological investigation of the lake physico-chemistry and the invertebrate fauna of Vaya Lake in 14 sites of the lake.[3] In this paper the results from processing of samples from eight selected sites are presented (sample points 1–5, 8, 9 and 11) – Figure 1. These eight sites were chosen also for the evaluation of the species composition of the phytoplankton [5] – the basis of the trophic webs – as most representative in reflecting the anthropogenic impact on the lake and are briefly described in the following.

The sites were visited by a speedboat and detected by means of GPS ‘GARMIN’. The rivers Aytoska, Chukarska and Sanardere are flowing in the western part of Vaya where the sampling point 1 (N42°29.655, E27°20.925) is situated. Against Gorno Ezerovo district sampling point 2 (N42°29.194, E27°22.085) is defined and the points 3 and 4 are located on the lake's central longitudinal axis (N42°30.160, E27°23.082 and N42°30.113, E27°24.518, respectively). Point 5 (N42°30.674, E27°25.013) is located against the canal discharging effluent of the urban wastewater treatment plant of Bourgas, point 8 (N42°29.665, E27°27.021) – against the channel, which connects the lake with the Black Sea, and point 9 (N42°29.458, E27°27.376) is in the channel itself. Point 11 (N42°30.750, E27°23.032) is situated opposite to Dolno Ezerovo district.

The following physico-chemical parameters were studied: water temperature (*T*, °C), pH, conductivity (EC, mS/cm), total alkalinity (Am, mE/L), free alkalinity (Ap, mE/L), total acidity (Kp, mE/L), dissolved oxygen (O_2_, mg/L) and oxygen saturation (sat%).[3,4] Since they serve as a background of the studied lake description, main results from them are mentioned here. Recently the lake has a β-mixooligohaline regime.[4] The temperature conditions were almost constant during the summer time (27 °C) with a slight increase in year 2005 (29.8 °C). Oxygen concentrations during the investigated period showed a significant fluctuation: 1.8 mg/L (October 2006) – 5.6 mg/L (August 2005). The pH was relatively high, reaching 9–9.8 in summer months (e.g., August 2005).[3]

The 1 litre samples were collected in glass bottles and fixed by 2%–4% formalin with further sedimentation in laboratory conditions. Forty-eight samples collected in August and October, 2004, 2005 and 2006 were processed in a standard way using Thoma blood-counting chamber on Carl Zeiss Jena Amplival microscope (magnification up to 500×) with the cell as a main counting unit. For biomass calculation in accordance with the cell shape geometrical formulae were used.[10,11] The standard structural parameters of the phytoplankton community – species diversity of Shannon–Weaver (*H*), evenness of Pielou (*E*) and dominance of Simpson (*c*) – were estimated.[12,13]

## Results and discussion

**Figure 2.**
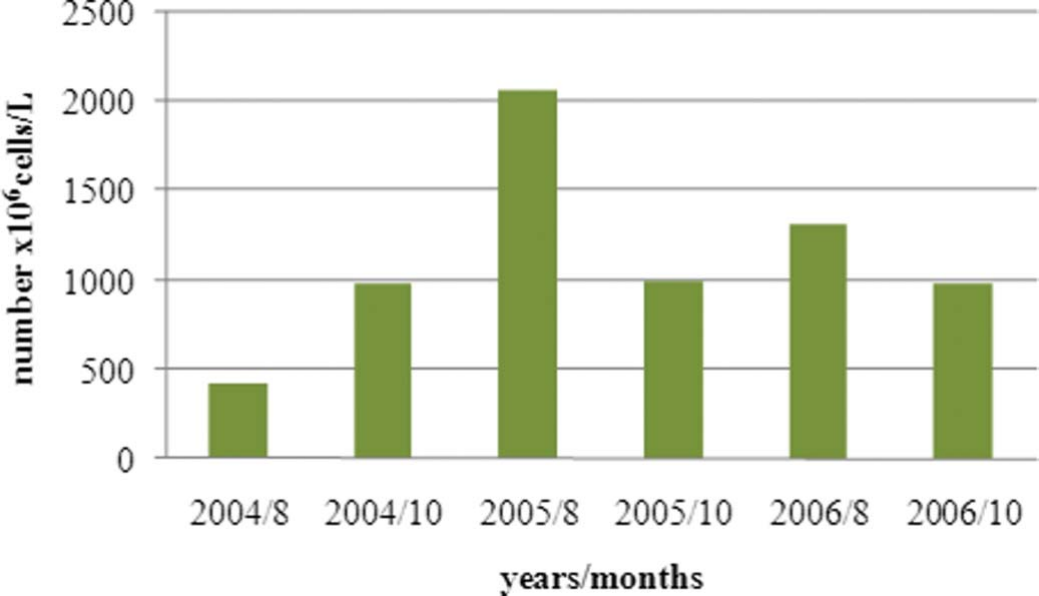
Dynamics of average total phytoplankton numbers in Vaya Lake (2004–2006): 8 August; 10 October.

The total phytoplankton numbers during the investigation period varied between 419 × 10^6^ cells/L and 2071.5 × 10^6^ cells/L. The minimum values were in August 2004, October 2004 and 2005, while the maximum numbers were in August 2005 and August 2006. The absolute mean value for the period was 1135 × 10^6^ cells/L (Figure 2).

**Figure 3.**
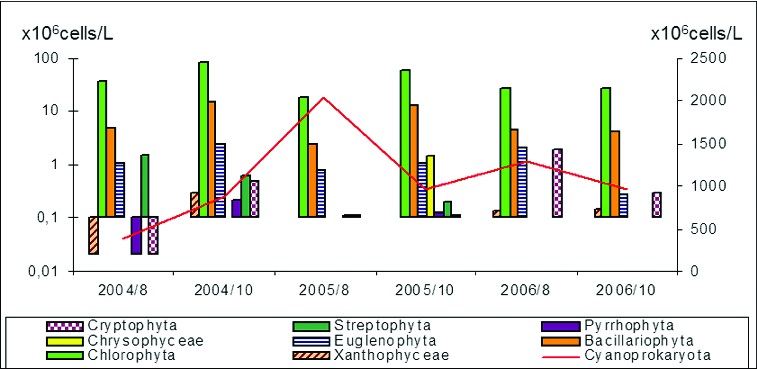
Average phytoplankton numbers of taxonomic groups in Vaya Lake (2004–2006). Red line is related with the right ordinate, which shows the numbers of Cyanoprokaryota.

**Figure 4.**
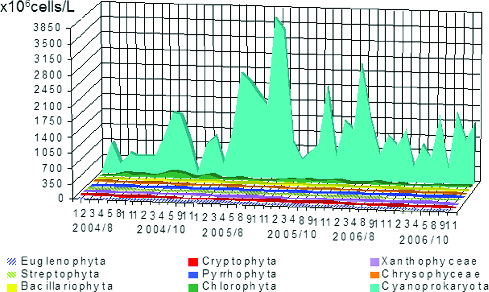
Dynamics of phytoplankton numbers of different taxonomic groups in sampling sites in Vaya lake (2004–2006).

**Figure 5.**
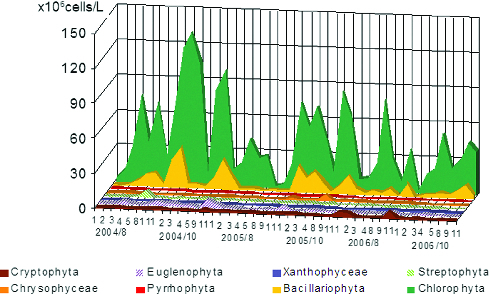
Dynamics of phytoplankton numbers of different taxonomic groups except Cyanoprakyorata in sampling sites in Vaya Lake by months of the studied years 2004–2006.

The maximum mean value of phytoplankton numbers (Figure 3) for all periods belonged to Cyanoprokaryota (1083.5 × 10^6^ cells/L), followed by Chlorophyta (42.6 × 10^6^ cells/L), Bacillariophyta (7.2 × 10^6^ cells/L) and Euglenophyta (1.3 × 10^6^ cells/L). Considerably less was the role of Streptophyta (0.61 × 10^6^ cells/L), Cryptophyta (0.50 × 10^6^ cells/L), Ochrophyta - Chrysophyceae (0.24 × 10^6^ cells/L) and Tribophyceae ( = Xanthophyceae – 0.10 × 10^6^ cells/L), and Pyrrhophyta (0.06 × 10^6^ cells/L). The dynamics of phytoplankton numbers during the studied years and their distribution in sampling sites is represented in Figures 4 and 5.

**Figure 6.**
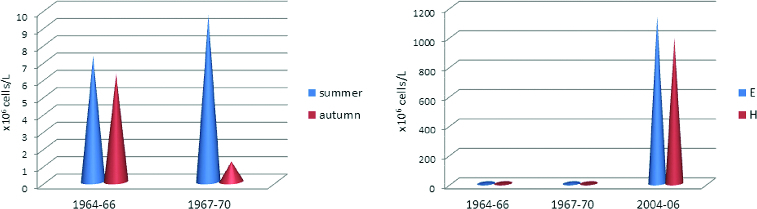
(A) The phytoplankton numbers in Vaya Lake for the summer and autumn during the first investigation periods (1964–1966 and 1967–1970) according to the data by Petrova (1967), Petrova-Karadjova (1974). (B) Comparison of the same results of the previous investigations with those obtained in our study (2004–2006).

In comparison to the detected abundance more than 30 years ago,[6,7] the phytoplankton numbers have risen 147 times for the summer period and up to 300 times for the autumn period (Figure 6). Similar estimations for the summer period were provided for the lake phytoplankton numbers by Pavlova et al. [9,14]

**Figure 7.**
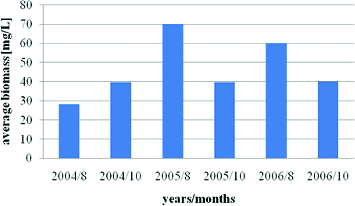
Dynamics of average total phytoplankton biomass in Vaya Lake (2004–2006): 8 August; 10 October.

The phytoplankton biomass in Vaya Lake varied between 27.13 and 69.5 mg/L. The maximum values of phytoplankton biomass were recorded during August months and had two peaks in August 2005 and August 2006 with obvious and significant increase when compared with the year 2004. The average value of biomas for all periods was 46 mg/L (Figure 7). Similar results were provided for the lake by Stoyneva [8], who pointed out 74.5 mg/L as the summer maximum values for 1995–2003 period, and by Pavlova et al. [9,14] for August 2004.

**Figure 8.**
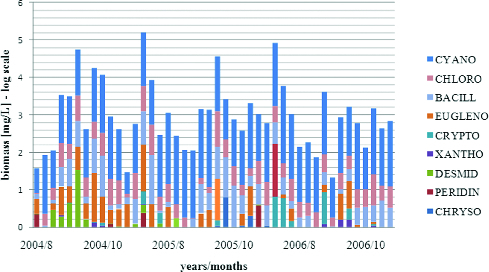
Phytoplankton biomass of taxonomic groups in sampling sites of Vaya Lake (2004–2006).

The biggest part of the total biomass always belonged to Cyanoprokaryota (Figure 8). The maximum biomass was always in August, with the highest value detected in the year 2005, when the highest number of cyanoporkaryote taxa was detected in sampling site 11. Bacillariophyta are on the second position as contributors to the total phytoplankton biomass. Their total values varied in the sampling sites and studied periods, and the highest values were during October 2005 and 2004 (points 1, 2 and 11). Euglenophyta take the third position with respect to their contribution for the total biomass. Their maximum was represented exclusively in October for three years of investigation, with the highest value estimated in October 2006 (point 1) and 2004 (point 11). Chlorophyta, Cryptophyta, Ochrophyta (Tribophyceae ( = Xanthophyceae) and Chrysophyceae), Pyrrhophyta and Streptophyta had lower contribution in the phytoplankton biomass.

Our data for the mean biomass value (46 mg/L) during the studied period allowed us to classify Vaya Lake as a hypereutrophic basin according to the open-boundary OECD system as it is compiled in Michev & Stoyneva.[2] They are strongly supported by the cyanoprokaryote dominance, which is generally known to attend the eutrophication events.[15] The strong phytoplankton development, leading to visible water blooms is supported by the high pH values (pH > 10), detected during the summer months, especially in sites 2 and 11 (see the text below).[3]

**Figure 9.**
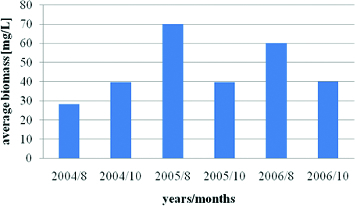
Dynamics of average phytoplankton carbon content in Vaya Lake (2004–2006); 8 August, 10 October.

The carbon content showed maxima in August 2005 and 2006 and minima in August 2004 (Figure 8). The mean value for the investigated period was 9.7 mg/L (Figure 9).

**Figure 10.**
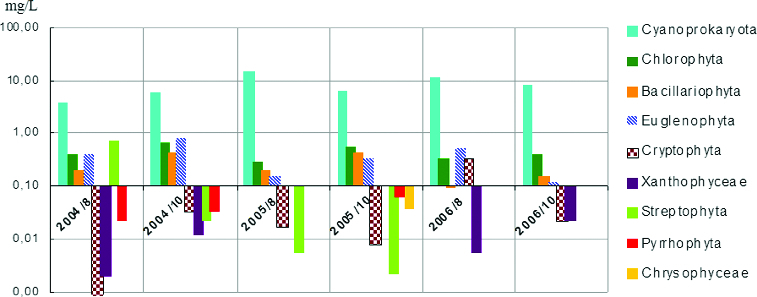
Average phytoplankton biomass of taxonomic groups in Vaya Lake (2004–2006).

Cyanoprokaryota dominated in the total carbon content of the phytoplankton with maximum value in August 2005 and August 2006 (Figure 10). The absolute maxima (25.1 и 26.9 mg/L) were detected in sampling points 2 and 11. Chlorophyta and Bacillariophyta were with the highest carbon content in October 2004 and October 2005. The highest values for both groups were in point 1. Euglenophyta contributed to the total phytoplankton carbon content with highest values in October 2004 and in August 2006 (maxima in points 1 and 11). The carbon content of Cryptophyta was with maxima in August 2006 in points 1, 2 and 11. The other groups had a low contribution to the total carbon content.

The results for the phytoplankton numbers, biomass and carbon content, which point the highest abundance and carbon values, as well as the highest contribution of indicative groups of cyanoprokaryotes and euglenophytes in sites 1, 2 and 11, clearly reflect the anthropogenic impact with organic pollution mainly from Dolno and Gorno Ezerovo districts. This is also supported by the data on the phytoplankton species composition, in which Cyanoprokaryota is second by species richness group after Chlorophyta during the same period [5] and eight years before.[8] Additional proof comes from the hydrochemical situation and the low oxygen content in site 1, in particular. There, during the summer, oxygen is almost lacking, leading to anaerobic conditions and appearance of sulphate reduction. In the same site, in winter and autumn, the oxygen content is the lowest for the lake. The results on conductivity also show the highest conductivity values in site 1 during the summer of 2004, related to the anaerobic processes and increase of hydrocarbonate and hydrosulphide ion concentration.[3]

**Figure 11.**
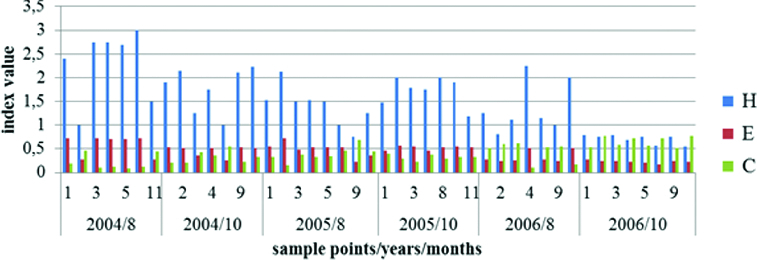
Dynamics of the biodiversity index (*H*), the indices of dominance (*c*) and evenness (*E*) in sampling sites in Vaya Lake (2004–2006).

The dynamics of the biodiversity index, the indices of dominance and evenness are shown in Figure 11. The values of biodiversity index (*H*) of Shannon–Weaver, for the investigated period varied from 0.55 (October 2006, point 11) to 3 (August 2004, point 8). The highest mean values of the biodiversity index were estimated for August 2004, followed by October 2005, and October 2004. A strong tendency for index decrease was detected in August and especially in October 2006.

The phytoplankton evenness index of Pielou (E) varied (from 0.17 to 0.78), keeping relatively high levels for the investigated period, but with a trend for decrease in 2006. High values for the evenness were calculated for August 2004 (sample points 1, 3, 5 and 8) and for August 2005 (sample point 2). The lowest evenness was registered in October 2006 (Figure 11).

The values of the coefficient of dominance of Simpson (*c*) varied from 0.09 (August 2004 in point 5) to 0.78 (October 2006 in point 11).

The strong decrease of species biodiversity index along with the increased coefficient of dominance and strong cyanoprokaryote dominance generally is known to accompany the change of the trophic level of different natural water basins with achievement of the eutrophic to hypertrophic status. For some work along these lines, see [16–20]. The same was proved for northern Bulgarian coastal lakes.[21] This statement corresponds fully with the results from our recent work, which demonstrate a decrease of biodiversity in the year 2006 with an increase of the coefficient of dominance (Figure 11).

## Conclusions

The data obtained on phytoplankton abundance (numbers, biomass and carbon content) with constant domination of Cyanoprokaryota clearly show that during the studied period Vaya Lake was a hypereutrophic basin. A trend of increase in the phytoplankton abundance has been established in comparison with previous phytoplankton studies, carried out around 30 years ago, as well as within the studied period from 2004 to 2006 [6–9,14] and is generally supported by the obtained hydrochemical results.[3] In this period, the values of the species diversity index and evenness decreased, while the values of the coefficient of dominance increased. The data collected reflect the total unfavourable direction of the wetland development and confirmed the site being declared earlier as a critically endangered one.[1,2] The proof of the extremely rapid increase of the abundance of the lake's primary producers could serve as an ‘alarm’ for starting the manangement activities for its restoration and conservation.
